# Persistent Tremor in Bipolar Disorder: A Case Report of Idiopathic Parkinson’s Disease Superimposed on Lithium and Antipsychotic Effects

**DOI:** 10.1155/crps/5580753

**Published:** 2026-02-06

**Authors:** Ethan Jetter, Daisy Valle, Diego Nolasco, Brent Carr

**Affiliations:** ^1^ College of Medicine, University of Florida, Gainesville, 32610, Florida, USA, ufl.edu; ^2^ Department of Psychiatry and Behavioral Sciences, Stanford University School of Medicine, Stanford, 94305, California, USA, stanford.edu; ^3^ Department of Psychiatry, University of Florida College of Medicine, Gainesville, 32610, Florida, USA, ufl.edu

**Keywords:** aripiprazole, bipolar disorder, drug-induced parkinsonism, lithium, Parkinson’s disease, quetiapine

## Abstract

We report a 58‐year‐old woman with bipolar I disorder on long‐term lithium and aripiprazole who developed a progressive asymmetric resting tremor and rigidity. Despite stopping both agents, the tremor persisted for more than a year. Dopamine transporter imaging showed reduced uptake in the left putamen, confirming idiopathic Parkinson’s disease (PD) with superimposed drug‐induced parkinsonism (DIP). Management included discontinuing lithium, switching aripiprazole to quetiapine to limit motor worsening, and starting carbidopa–levodopa. Motor symptoms improved, but hypomanic symptoms emerged and required psychiatric dose adjustments, while apathy remained prominent. The case illustrates diagnostic overshadowing in bipolar disorder (BD) and highlights two practical lessons. When parkinsonian signs are atypical or persist after medication changes, consider idiopathic PD rather than attributing symptoms to side effects. Care is best delivered through close collaboration between psychiatry and neurology to balance dopaminergic therapy with mood stabilization.

## 1. Introduction

Epidemiological evidence indicates that patients with bipolar disorder (BD) have a three‐ to sixfold higher risk of receiving a Parkinson’s disease (PD) diagnosis compared to the general population [1–3]. Despite this association, parkinsonian symptoms in psychiatric patients are often attributed to medication side effects, potentially delaying the diagnosis of underlying neurodegenerative disease [4, 5]. Lithium typically causes a fine symmetric postural or action tremor but can rarely precipitate parkinsonism, and antipsychotics frequently induce parkinsonism making it challenging to distinguish drug‐induced symptoms from idiopathic PD [6, 7]. Whether the BD–PD association reflects shared pathophysiology or confounding by drug‐induced parkinsonism (DIP) is not fully understood [8]. However, in a prospective cohort study of over 500,000 participants, BD patients had higher rates of developing PD compared to those without BD, and this association persisted in both sensitivity analyses excluding patients on lithium, antiepileptics, or antipsychotics at enrollment and mediation analyses finding BD independently predicted PD even after accounting for patient use of these medications at enrollment, suggesting that factors beyond these medications may contribute to the increased rate of PD in BD patients [9]. We present a case where persistent asymmetric parkinsonian features initially attributed to lithium and aripiprazole ultimately revealed underlying idiopathic PD, highlighting the phenomenon of diagnostic overshadowing in psychiatric populations.

## 2. Case Presentation

Ms. A was a 58‐year‐old woman with bipolar I disorder maintained on lithium 450 mg twice daily and lamotrigine 200 mg twice daily for long‐term mood stabilization. Six years prior to presentation, she underwent a sleep evaluation for chronic insomnia, loud snoring, restless leg symptoms, vivid dream enactment, and frequent sleeptalking. Polysomnography confirmed moderate obstructive sleep apnea, and a diagnosis of restless legs syndrome (RLS) was made based on clinical features and a low ferritin level (36 ng/mL). Iron supplementation and continuous positive airway pressure (CPAP) therapy were initiated, though her adherence was inconsistent.

Nine months later, Ms. A developed a noticeable right‐sided resting hand tremor, which her outpatient psychiatry team attributed to chronic lithium therapy. She continued her medications without change at this time. Nearly 3 years later, she was hospitalized for worsening tremulousness, gait instability, and confusion. Lab tests confirmed lithium toxicity (serum lithium 2.1 mmol/L) accompanied by acute kidney injury (creatinine 1.16 mg/dL, baseline ~0.8). The consultation‐liaison (C‐L) psychiatry service recommended holding lithium until renal function recovered. Lithium was then cautiously resumed; however, 4 months later, Ms. A was readmitted with disorientation, a coarse hand tremor, and hyperreflexia. At this time, her lithium level was again elevated at 2.0 mmol/L, resulting in a diagnosis of recurrent lithium toxicity. The C‐L team advised permanently discontinuing lithium due to repeated toxicity. Lamotrigine was continued at 200 mg twice daily, and aripiprazole 5 mg daily was started to further stabilize her mood. At subsequent outpatient psychiatry follow‐up visits, her resting hand tremor was noted to be unchanged from prior visits, despite lithium discontinuation.

Fourteen months after lithium discontinuation, Ms. A was referred to outpatient neurology for evaluation of worsening short‐term memory, ongoing vivid dream enactment, and a persistent tremor. A dopamine transporter SPECT scan (DaTscan) demonstrated asymmetric decreased tracer uptake in the left putamen. While the scan favored idiopathic PD, the symptom chronology and dopamine‐antagonist exposure suggested DIP. Neurology diagnosed idiopathic PD with superimposed DIP, most likely from aripiprazole. Two months later, neurology initiated carbidopa–levodopa 25/100 mg three times daily for her motor symptoms and recommended discontinuation of aripiprazole. At psychiatric follow‐up 2 months later, aripiprazole was tapered off, and quetiapine 200 mg nightly was started for mood stabilization, as a safer antipsychotic choice for a patient with PD.

Over the following months, Ms. A’s motor symptoms improved on carbidopa–levodopa, but she developed marked apathy and low motivation. Venlafaxine XR was titrated to 150 mg twice daily to address her depressive and apathetic symptoms. Because this dosage offered little relief, bupropion XL was subsequently added and titrated to 300 mg daily. These changes had minimal impact on her apathy or mood, although her parkinsonian motor signs remained well‐controlled.

**Figure 1:**
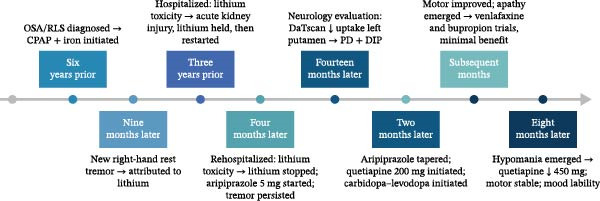
Timeline of key clinical events in Ms. A’s case. CPAP, continuous positive airway pressure; DIP, drug‐induced parkinsonism; OSA, obstructive sleep apnea; RLS, restless legs syndrome. PD, idiopathic Parkinson’s disease.

Eight months after starting carbidopa‐levodopa, Ms. A began to experience reduced need for sleep, pressured speech, irritability, and increased goal‐directed activity, symptoms concerning for emerging hypomania. In response, her quetiapine dose was gradually titrated up to 450 mg nightly. At her last follow‐up, her tremor and rigidity were stable on carbidopa–levodopa, but Ms. A continued to report significant apathy and intermittent mood fluctuations. A timeline of key clinical events is presented in Figure 1.

## 3. Discussion

When a patient with BD develops a tremor or parkinsonian features, the reflex assumption is often that it is a medication side effect. Lithium, antipsychotics, and valproate all commonly produce tremors or parkinsonian features. In Ms. A’s case, two clear episodes of lithium toxicity made a drug‐induced cause especially compelling; indeed, lithium can, in rare instances, precipitate parkinsonism beyond its usual coarse action tremor, but typically, lithium causes a fine symmetric postural or action tremor rather than a resting tremor [6]. Even in toxicity, the tremor may become coarser but generally remains action‐predominant rather than a true resting tremor [10, 11]. Thus, an asymmetric resting tremor persisting for over a year after stopping lithium and tapering aripiprazole was atypical for a drug effect and more consistent with idiopathic PD. The key distinguishing features of idiopathic PD, DIP, and lithium tremor are summarized in Table 1.

**Table 1:** Clinical features of idiopathic Parkinson’s disease, drug‐induced parkinsonism, and lithium‐induced tremor.

Domain	Idiopathic Parkinson’s disease (PD)	Drug‐induced parkinsonism (DIP)	Lithium‐induced tremor
Onset and timing	Gradual, insidious onset in mid‑late life [12]	Days–weeks after starting or increasing a dopamine‑blocking medication [7]	Early in treatment; dose‑related [10]
Symmetry	Usually asymmetric (often one side) [13]	Variable; classically symmetric but asymmetry frequent [7, 14]	Symmetric [10]
Tremor type	Rest tremor; pill‑rolling quality [12]	Tremor variable; rigidity and bradykinesia predominate	Fine postural or action tremor [6]
Response to withdrawal	No improvement after dose reduction or medication discontinuation [12]	Improves after stopping the causative drug [7]	Improves with dose reduction or medication discontinuation [10]
DaTscan imaging	Reduced striatal uptake (nigrostriatal degeneration) [13]	Normal striatal uptake (intact presynaptic neurons) [13]	Normal uptake [6]
Prodromal features	^a^Nonmotor prodrome common [12]	None; develops only after drug exposure [7]	None; tremor is a pharmacologic effect [10]

^a^Nonmotor prodrome features commonly include rapid eye movement sleep behavior disorder, hyposmia, and constipation [12].

Distinguishing DIP from idiopathic PD is often impossible at the bedside [14]. Both syndromes share bradykinesia, rigidity, and often tremor. However, DIP typically, develops within weeks to months of antipsychotic exposure, and usually improves within weeks to months after the offending drug is withdrawn, though recovery may occasionally take over a year [7, 15]. Rarely, symptoms persist indefinitely, which has been termed “tardive parkinsonism” [7, 16]. While DIP has classically been described as symmetric, asymmetry has been observed in 20.8%−53.8% of cases across various studies, rendering symmetry unreliable for differentiating DIP from idiopathic PD [14, 17–20]. Idiopathic PD, in contrast, often starts insidiously and asymmetrically, with one side of the body more affected [21, 22]. Unlike DIP, which typically resolves after drug withdrawal, PD symptoms characteristically persist and progress overtime [14, 20]. In Ms. A’s case, the continued presence of an asymmetric resting tremor and progression of symptoms even after stopping lithium and aripiprazole pointed toward underlying PD rather than pure DIP.

Ms. A’s history of sleep disturbances provided additional diagnostic clues. Years before her PD diagnosis, she suffered from insomnia, vivid dream enactment, and talking in her sleep, features highly suggestive of undiagnosed rapid eye movement sleep behavior disorder (RBD). In fact, when neurology evaluated her, they noted that Ms. A “probably” had RBD based on her history, though her prior polysomnography report did not include assessment for REM sleep without atonia, the diagnostic polysomnographic finding for RBD [23]. RBD is an established prodromal marker of PD and is associated with a more malignant course, predicting worse motor outcomes and a heavier burden of nonmotor symptoms such as apathy and cognitive impairment [24, 25]. Additionally, she had long‐standing RLS, which has been linked to a higher short‐term risk of developing PD [26]. Together, the presence of probable RBD and longstanding RLS strengthens the interpretation that her parkinsonism was not simply drug‐induced, but part of an idiopathic PD process.

Ancillary testing can strengthen diagnostic certainty when clinical features are ambiguous. Dopamine transporter imaging is the most reliable tool for distinguishing DIP from degenerative PD. In pure DIP, the presynaptic dopaminergic neurons are generally intact, so the DaTscan shows normal tracer uptake in the striatum. In idiopathic PD, by contrast, there is loss of striatal dopaminergic neurons, and the DaTscan shows reduced uptake, especially in the posterior putamen, often asymmetrically [7, 13, 27, 28]. DaTscan and related techniques consistently show reduced dopaminergic uptake in PD, but normal uptake in DIP, with high specificity for underlying neurodegeneration [29]. In Ms. A’s case, the DaTscan revealed asymmetric dopaminergic loss in the left putamen, confirming underlying neurodegeneration and supporting the diagnosis of idiopathic PD with superimposed DIP. Beyond dopamine transporter imaging, other diagnostic modalities such as MIBG scintigraphy, transcranial ultrasound, and skin biopsy for alpha‐synuclein can also help distinguish idiopathic PD from DIP [20].

The diagnosis of PD is often missed or delayed in psychiatric patients due to diagnostic overshadowing. When a patient on long‐term psychotropics develops tremor, slowness, or rigidity, clinicians often reflexively attribute these signs to medication side effects. Such assumptions can obscure an emerging primary neurologic disorder. In Ms. A’s case, her history of lithium toxicity and aripiprazole use initially reinforced a DIP narrative, contributing to a delay in recognizing the idiopathic PD developing underneath. She experienced genuine lithium and aripiprazole‐induced parkinsonism that clouded the clinical picture, and only with time and a DaTscan did the true PD become evident.

Ms. A’s clinical course illustrates the therapeutic “dopamine seesaw” in managing co‐occurring PD and BD: every adjustment to improve motor symptoms risks destabilizing her mood. Initiation of carbidopa–levodopa significantly relieved her rigidity and tremor, but shortly thereafter, she developed hypomanic symptoms. Dopamine replacement therapy is well known to influence mood, and subsyndromal hypomanic or manic symptoms are relatively common on dopaminergic therapy: nearly half of PD patients with motor fluctuations have been observed to experience subthreshold hypomania, which typically resolves when the dopaminergic dose is lowered [30]. Full‐blown mood episodes are less frequent, but still clinically significant: approximately 6% of patients may develop mania and 11% hypomania in the course of dopamine replacement therapy for PD [31]. Moreover, long‐term dopaminergic treatment can produce nonmotor fluctuations that manifest as abrupt, transient shifts in energy, affect, or motivation, which may mimic or exacerbate underlying mood instability in a patient with comorbid BD [32]. Ms. A’s episode of hypomania following the uptitration of carbidopa–levodopa underscores the importance of vigilant monitoring of mood and behavior whenever dopaminergic therapy is initiated or increased in a patient with mood‐disorder vulnerability. Clinicians should frequently reassess mood during PD medication titrations to catch early signs of hypomania or mania.

Treating Ms. A’s BD became more complex once PD entered the picture, because the usual first‐line agents for bipolar mania, antipsychotics can worsen parkinsonian motor symptoms. Her trial of aripiprazole, a partial *D*_2_ agonist often used in BD, paradoxically exacerbated her PD features by worsening rigidity and bradykinesia. Although aripiprazole’s partial *D*_2_ agonism might suggest a lower risk of parkinsonism, in practice, it can still precipitate significant extrapyramidal symptoms. This outcome aligns with evidence from open trials and case reports that aripiprazole can cause significant motor deterioration in patients with PD, and even trigger parkinsonism in individuals without PD [33–35]. After aripiprazole was discontinued, quetiapine was selected for mood stabilization due to its minimal dopamine *D*_2_ blockade [36]. However, quetiapine’s efficacy in PD psychosis is uncertain; some trials showed no advantage over placebo, and it may be less effective than pimavanserin or clozapine [37, 38]. Expert consensus holds that clozapine and pimavanserin have the strongest evidence for managing PD psychosis without exacerbating motor symptoms, although clozapine’s use is limited by the risk of agranulocytosis and the need for regular blood monitoring [38–40]. Whereas quetiapine’s benefit is less clear, and it should generally be reserved for cases where clozapine or pimavanserin are not feasible [37, 38, 40, 41].

**Figure 2:**
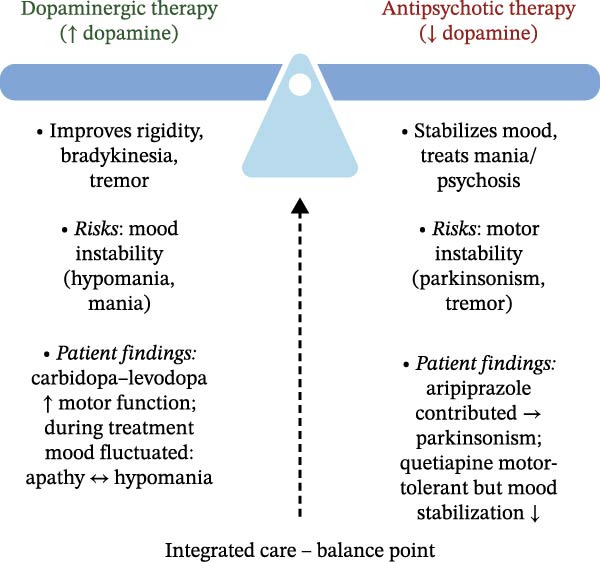
Therapeutic balance between dopaminergic and antipsychotic therapies. The “dopamine seesaw” illustrates the therapeutic balance between dopaminergic therapy, which increases dopamine to improve Parkinsonian symptoms, and antipsychotic therapy, which decreases dopamine to stabilize mood. The balance point represents the coordinated efforts of the neurologist, outpatient psychiatrist, and consultation‐liaison psychiatry service to optimize motor function and mood stability throughout treatment.

In Ms. A’s case, it is noteworthy that antipsychotics were being used primarily for mood stabilization rather than for psychosis. Her inability to tolerate aripiprazole and her subsequent stabilization on quetiapine illustrate the challenge of selecting antipsychotics in PD, where *D*_2_ receptor activity must be balanced against both psychiatric efficacy and motor tolerability. At the same time, pimavanserin is increasingly favored over traditional dopamine‐blocking antipsychotics when treating hallucinations or delusions in PD psychosis [41, 42]. As a selective 5‐HT_2_A inverse agonist without dopamine receptor affinity, pimavanserin is thought to carry a lower risk of motor side effects but lacks the dopaminergic mechanisms implicated in antimanic efficacy [40]. Notably, pimavanserin is FDA–approved only for PD psychosis and has not demonstrated efficacy for other psychiatric indications [43, 44]. Thus, for Ms. A’s psychiatric symptoms, which stemmed from BD rather than PD psychosis, pimavanserin would not be an appropriate choice for treatment. Ms. A’s outcome reinforces the principle that, in patients with comorbid PD and BD, antipsychotic selection should prioritize motor tolerability, and taken together, her management highlights the therapeutic tension between dopaminergic therapy to relieve motor symptoms and antipsychotic therapy to stabilize mood. This therapeutic balance is depicted in Figure 2.

Ms. A’s depressive symptoms and profound apathy proved challenging to treat. Trials of venlafaxine and bupropion yielded little improvement in her motivation or mood. In general, antidepressants can be beneficial for mood symptoms in PD, and recent meta‐analytic data indicate that newer‐generation antidepressants such as SSRIs, SNRIs, and related agents reduce depressive and anxiety symptoms in PD without serious adverse effects, though conclusions are limited by small sample sizes and few available trials [45]. However, clinical trials have shown variable efficacy, and no single antidepressant has emerged as clearly superior for PD–related depression [46]. It is also important to consider the influence of dopaminergic therapy on mood: dopamine agonist use is associated with lower motivational symptoms such as apathy and anhedonia in PD, but was not found to be associated with core depressive symptoms like sadness or hopelessness [47]. This suggests that some of Ms. A’s apathy might respond better to enhancing dopaminergic stimulation, although in her case, any dose increases were limited by the risk of precipitating hypomania.

In patients with BD, any use of antidepressants requires caution due to the risk of precipitating mania or mood switching. SNRIs such as venlafaxine are known to carry a higher risk of mood switching into hypomania or mania [48]. In contrast, bupropion is generally considered to have a lower risk of inducing mania and has the added theoretical benefit of dopaminergic activity, which might improve apathy. Indeed, small studies and case reports in PD suggest that bupropion may alleviate depressive symptoms and apathy, even when other antidepressants have failed [49, 50]. It is crucial, of course, that any antidepressant in a BD patient be paired with a mood stabilizer to mitigate mood switching; appropriately, Ms. A remained on lamotrigine throughout her antidepressant trials. The movement disorder society’s evidence‐based reviews have rated antidepressant therapy as “clinically useful” for depression and apathy in PD [12]. Yet despite these evidence‐based interventions, Ms. A’s response was minimal, illustrating how depression and apathy often remain refractory in such complex cases. In addition to medications, clinicians may also consider nonpharmacologic measures such as neuropsychological evaluation, occupational or physical therapy and structured exercise programs to address apathy, cognitive decline and safety concerns [51]. Her outcome highlights the importance of close collaboration between neurology and psychiatry, cautious titration of both dopaminergic and psychotropic medications, and active involvement of the patient and family in the treatment plan. Managing co‐occurring PD and BD is a dynamic process, with the “dopamine seesaw” requiring constant recalibration to maintain both motor function and mood stability.

## 4. Conclusion

In patients with BD, parkinsonian symptoms are frequently attributed to the side effects of lithium or antipsychotic medications. This case illustrates that such an assumption, while common, can lead to diagnostic delays. Ms. A’s tremor and rigidity were initially explained away by lithium toxicity and antipsychotic exposure, yet the persistence of an asymmetric resting tremor despite stopping those medications proved to be idiopathic PD. An abnormal dopamine transporter scan ultimately confirmed underlying neurodegeneration, unmasking PD that drug effects had obscured, a clear example of diagnostic overshadowing.

Even with careful adjustments, some of Ms. A’s symptoms, particularly apathy and low motivation, remained difficult to treat, highlighting the limitations of current therapies for such complex cases. Optimal care in such dual‐diagnosis cases hinges on an integrated, interdisciplinary approach, ensuring timely recognition of neurological disease in psychiatric patients and helping to craft a balanced treatment plan that addresses both motor and mental health. In summary, clinicians should maintain a high index of suspicion for PD in BD patients with atypical or persistent tremors and should strive for collaborative management that navigates the competing needs of both conditions.

## Funding

This research received no specific grant from any funding agency in the public, commercial, or not‐for‐profit sectors.

## Consent

Written consent was not available from the patient. From the manuscript, we have omitted any nonessential identifiers and information related to the identity of the patient to sufficiently anonymize the patient in accordance with ICMJE guidelines.

## Conflicts of Interest

The authors declare no conflicts of interest.

## Data Availability

Data sharing is not applicable to this article as no datasets were generated or analyzed during the current study.
